# Effects of Blueberry and Collard Greens Dietary Intervention on Metabolic and Inflammatory Biomarkers in Zucker Obese Rats

**DOI:** 10.3390/nu18193246

**Published:** 2026-10-01

**Authors:** Jacqueline A. Yeboah, Chastity N. Bradford, Norma L. Dawkins

**Affiliations:** 1 Department of Food and Nutritional Sciences, Tuskegee University, 1200 W. Montgomery Rd, Tuskegee, AL 36088, USA; ndawkins@tuskegee.edu; 2 Department of Biology, Tuskegee University, 1200 W. Montgomery Rd, Tuskegee, AL 36088, USA; cbradford@tuskegee.edu

**Keywords:** obesity, cardiovascular diseases, blueberries, collard greens, blood glucose, lipid profile, inflammation, Zucker rats

## Abstract

**Background/Objectives:** Obesity is a global health challenge associated with metabolic and inflammatory health outcomes. Dietary interventions such as phytochemical-rich foods may offer a sustainable and cost-effective approach to improving health outcomes associated with obesity. This study evaluated the effects of diets containing 4% blueberry powder and extracts, and 4% collard greens powder and extracts, and a combination of 2% blueberry and 2% collard greens on metabolic and inflammatory biomarkers in obese Zucker rats. **Methods**: Fifty-six male rats were randomly assigned to seven treatment groups (*n* = 8), including five diet treatment groups, an AIN- 93 control group, and an Ozempic^®^ (semaglutide)-treated group. Body weight, feed intake, plasma glucose, lipid profile, and inflammatory biomarkers were assessed following an 8-week dietary intervention. **Results**: A high mortality rate was observed in the Ozempic^®^ (semaglutide)-treated group, with five out of eight rats dying during the third week of intervention. The semaglutide-treated group showed the greatest reductions in fasting plasma glucose concentrations. Diets supplemented with blueberry and collard greens significantly increased HDL cholesterol and reduced LDL cholesterol concentrations compared with the control group, while also improving selected inflammatory biomarkers. The collard green extract group had the lowest IL-6 concentration (34.65 ± 3.90 pg/mL) compared with the control group (339.57 ± 185.65 pg/mL) (*p* < 0.05). **Conclusions**: Overall, blueberries and collard greens consumption improved dyslipidemia and inflammation in obese Zucker rats. These preliminary findings suggest that phytochemical-rich fruits and vegetables may provide a cost-effective dietary strategy for improving metabolic and inflammatory outcomes associated with obesity.

## 1. Introduction

Obesity is a public health concern linked to chronic diseases such as diabetes, cancer, and stroke [1,2]. Despite several efforts to curb obesity, its prevalence continues to rise [3]. Reports by the World Health Organization indicate that as of 2022, 43% of adults aged 18 and older were overweight, while approximately 16% were classified as obese (WHO, 2025). These increasing rates are attributed to factors such as a lack of physical activity and poor dietary habits, including high intake of processed foods and low intake of fruits and vegetables [4,5,6]. Obesity is associated with metabolic complications, including impaired glucose regulation, dyslipidemia, and chronic low-grade inflammation [7,8,9]. These metabolic disturbances increase an individual’s risk of developing cardiovascular disease [9].

The inclusion of fruit and vegetables in the diet has consistently been associated with a reduced risk of chronic diseases [10,11]. Studies have shown that fruits and vegetables are rich sources of fiber, minerals, vitamins, and bioactive compounds that influence satiety, fat storage, metabolism, and inflammation [12,13]. Bioactive compounds such as polyphenols, saponins, terpenoids, and flavonoids in fruits and vegetables possess antioxidant, anti-inflammatory, and lipid-lowering effects that may contribute to the management of metabolic and inflammatory complications [14,15]. Consequently, fruits and vegetables are used as therapeutic foods for the management of chronic diseases [16].

Among different fruit species, blueberries have gained attention for their nutrient and bioactive profiles [17,18]. They are rich in phenolic compounds which contribute to their health promoting properties [19,20]. They also contain high amounts of fiber, minerals, and organic acids, which are essential for health [21,22]. Studies have reported that blueberries contain fifteen types of anthocyanins and five types of cinnamic acid derivatives responsible for their biological activity [20]. Additionally, studies have demonstrated that blueberry consumption reduces low-density lipoprotein (LDL) levels and blood pressure [23,24]. The fiber and phytochemicals in blueberries slow down digestion and improve satiety [25].

Similarly, collard greens are a cruciferous vegetable rich in fiber, minerals, and amino acids including leucine, lysine, methionine and histidine [26]. Glucosinolates are the predominant bioactive compound in collard greens responsible for its anti-inflammatory properties [27,28]. Collard greens also contain high amounts of flavanols and phenolics, including kaempferol and quercetin, which reduce oxidative stress [29].

Blueberries have been extensively investigated for their potential cardiometabolic benefits; however, most studies have focused on their effects as a single dietary component. Similarly, while collard greens contain bioactive compounds such as glucosinolates, their role in improving metabolic and inflammatory dysfunction associated with obesity remains less explored. Furthermore, limited research has investigated the comparative effects of individual and combined formulations of blueberries and cruciferous vegetables on metabolic and inflammatory dysfunction of obesity.

Therefore, we hypothesized that diets containing blueberries, collard greens, or their combination would influence metabolic and inflammatory markers associated with obesity. This study aimed to evaluate the effects of these dietary interventions on selected metabolic and inflammatory biomarkers, with body weight monitored throughout the experimental period. Ozempic^®^ (semaglutide) was included as a pharmacological positive control. Powder and extract formulations of blueberry and collard greens were incorporated into the experimental diets to determine whether their concentrated extracts produced comparable or differential effects relative to their respective powder formulations.

## 2. Materials and Methods

### 2.1. Diet Formulations

Both freeze-dried powders and extracts of wild blueberries and collard greens were used in this study. Freeze-dried wild blueberry powder and freeze-dried collard greens powder were purchased from Z-Natural Foods, West Palm Beach, FL, USA, and North Bay Trading Company, Brule, WI, USA, respectively. Extracts were prepared in accordance with the method described by [30].

Briefly, 100 g of blueberry powder and 100 g of collard greens powder were separately mixed with 1 L of 96% ethanol, corresponding to a 1:10 (*w*/*v*) solid-to-solvent ratio. The mixtures were covered with aluminum foil and incubated in the dark at 25 °C for 48 h [30]. Following incubation, the mixtures were filtered using filter paper to separate the solid plant material from the ethanol-containing filtrates. The resulting filtrates were collected and concentrated using a rotary evaporator for 1 h to remove the ethanol and obtain concentrated blueberry and collard greens extracts. The extraction yield of each extract was calculated as the mass of extract recovered relative to the initial mass of freeze-dried powder used, using the following formula:

$$\mathrm{Extraction}\;\mathrm{yield}\;=\;\frac{mass\;of\;recovered\;extract}{initial\;mass\;of\;freeze\;dried\;powder\;}\;\times 100\%$$

The extraction resulted in extraction yields of 21% for blueberry extract and 26% for collard greens extract. The powders and their corresponding extracts were subsequently submitted to Research Diets, Inc. (New Brunswick, NJ, USA) for the formulation of experimental diets and determination of their nutritional composition.

The experimental diets formulated by Research Diets consisted of modified AIN-93 with (a) 4% blueberry powder, (b) 4% blueberry extract, (c) 4% collard greens powder, (d) 4% collard greens extract and (e) a combination 2% blueberry powder and 2% collard greens powder. AIN-93 served as control diet for the experiment. The nutritional composition of experimental diets is listed in Table 1.

### 2.2. Animal Care and Use

The Tuskegee University Animal Care and Use Committee (Tuskegee, AL 36088, USA) approved the protocol for this research study, in accordance with standards established by the National Institutes of Health. The protocol number for this research is R 15-2025-09.

### 2.3. Experimental Design

Sixty five-week-old male obese-prone Zucker rats (fa/fa), weighing 70–100 g, were obtained from Charles River Laboratories, Inc. (Wilmington, MA, USA). Upon arrival at the Tuskegee University College of Veterinary Medicine Animal Care Facility, the rats were housed two per cage at a temperature of 21 ± 1 °C, a relative humidity of 50 ± 5%, and a 12 h light/12 h dark cycle. The rats were acclimatized for two weeks, during which they had ad libitum access to standard rodent chow and water.

Following the two-week acclimatization period, 56 rats were weighed and stratified into two categories based on baseline body weight (heavy and light). Animals within each stratum were randomly allocated across the seven experimental groups (*n* = 8 per group). Rats were initially housed two per cage, resulting in four cages per treatment group. The remaining four rats were not included in the study and were not assigned to any treatment group. A sample size of eight rats per treatment group was selected based on previous dietary and nutritional intervention studies involving Zucker rats [31,32]. Based on these studies, eight rats per group were considered sufficient to detect biological responses while minimizing animal use. Based on this precedent, eight rats were allocated to each experimental group.

The experimental groups consisted of five dietary intervention groups, a control group, and a positive control group, as follows:

a. Male Zucker rats receiving modified AIN-93 with 4% blueberry powder.
b. Male Zucker rats receiving modified AIN-93 with 4% blueberry extract.
c. Male Zucker rats receiving modified AIN-93 with 4% collard greens powder.
d. Male Zucker rats receiving modified AIN-93 with 4% collard greens extract.
e. Male Zucker rats receiving modified AIN-93 with 2% collard greens powder and 2% blueberry powder.
f. Male Zucker rats receiving the standard AIN-93 diet, serving as the control group.
g. Male Zucker rats receiving the standard AIN-93 diet with an additional pharmacological intervention of Ozempic^®^ (semaglutide) serving as the positive control.

Ozempic^®^ (semaglutide) was included as a pharmacological benchmark for the dietary interventions because of its well-established effects on body weight and metabolic outcomes, including glucose regulation and inflammation [33,34]. The commercially available Ozempic^®^-prefilled pen (8 mg/3 mL; 2.68 mg/mL), manufactured by Novo Nordisk A/S, DK-2800 Bagsvaered Denmark, was used for this study. The drug was administered directly from the pen without dilution using a syringe. A dosage of 0.25 mg/kg body weight was assigned to the positive control group once per week for 8 weeks, as described in a previous study by [35]. Doses of the drug were administered by subcutaneous injection, as described by [35]. The injection volume of semaglutide was calculated for each rat based on individual body weight, the prescribed dose (0.25 mg/kg body weight), and the semaglutide concentration (2.68 mg/mL). The injection volume (mL) was calculated using the formula injection volume (mL) = $\;\frac{dose\;(mg/kg)\times body\;weight\;(kg)}{drug\;concentration\;(mg/mL)}$.

The dietary intervention lasted for 8 weeks, during which rats had ad libitum access to their respective experimental diets and water. Body weight was recorded at baseline and weekly throughout the intervention. Feed intake was recorded daily at the cage level, with the cage serving as the experimental unit. Daily feed intake for each cage was determined as the difference between the amount of feed provided and the amount remaining. Daily energy intake for animals in each cage was subsequently calculated by multiplying the amount of feed consumed by the energy density of the corresponding experimental diet.

Rats were monitored daily for clinical signs of illness and dehydration. Any abnormal observations were reported to both the veterinary staff and the Institutional Animal Care and Use Committee (IACUC). All animals were maintained and handled under the same routine care conditions until the third week, when five of the eight rats in the Ozempic-treated group died following observed reductions in food intake and signs of dehydration. Following the deaths, the three surviving rats in the Ozempic-treated group were housed individually, resulting in three cages with one rat per cage. Thereafter, feed and energy intake for the Ozempic-treated group were recorded separately for each of the three cages.

Following veterinary guidance, the surviving rats received approximately 9–15 mL of fluids subcutaneously 2 h after each weekly Ozempic administration for the remainder of the study. Supportive fluid was administered only to the surviving rats in the Ozempic-treated group. All other treatment groups continued under the same routine care conditions without supportive fluids. Cage cleaning and bedding changes were performed twice weekly throughout the experimental period to maintain proper animal husbandry and hygiene.

At the end of the intervention, rats were fasted overnight and euthanized by carbon dioxide (CO_2_) inhalation. To minimize pain and distress, euthanasia was performed while the rats were under anesthesia. Death was confirmed by the absence of a heartbeat and respiration. After confirming death, whole blood (10 mL) was collected immediately by terminal cardiac puncture into EDTA-treated tubes and centrifuged at 1500× *g* for 15 min at 4 °C. Plasma was separated and stored at −80 °C until analysis. Plasma samples were used to measure fasting glucose, lipid profiles, and inflammatory biomarkers.

### 2.4. Blood Glucose

Plasma glucose concentrations were measured using the Crystal Chem Glucose Assay Kit (Catalog 81693). Concentrations were determined according to the manufacturer’s instructions. The assay had a measurement range of 0–675 mg/dL. Samples with glucose concentrations exceeding 675 mg/dL were diluted 1:2 with the appropriate diluent and reanalyzed. The measured concentrations were multiplied by the corresponding dilution factor to obtain the final glucose concentrations.

### 2.5. Lipid Profile

Plasma LDL cholesterol (LDL-C) concentrations were measured using the Crystal Chem LDL-Cholesterol ELISA Kit (Catalog No. 79960) according to the manufacturer’s instructions. The assay had a measurement range of 4.5–245 mg/dL. Samples with LDL-C concentrations exceeding 245 mg/dL were diluted 1:2 with the appropriate diluent and reanalyzed. The measured concentrations were multiplied by the corresponding dilution factor to obtain the final LDL-C concentrations.

Plasma HDL cholesterol (HDL-C) concentrations were measured using the Crystal Chem HDL-Cholesterol ELISA Kit (Catalog No. 7990) according to the manufacturer’s instructions. The assay had a measurement range of 1.5–180 mg/dL. Samples with HDL-C concentrations exceeding 180 mg/dL were diluted 1:2 with the appropriate diluent and reanalyzed. The measured concentrations were multiplied by the corresponding dilution factor to obtain the final HDL-C concentrations.

Total cholesterol was determined according to the manufacturer’s protocol using a Rat Cholesterol Assay Kit (TSZ ELISA, Catalog No. R6955), with a manufacturer-specified assay range of 15–400 U/L. Cholesterol concentrations were determined from the standard curve and expressed in mmol/L. No sample dilution was required for this analysis. Cholesterol concentrations were subsequently converted from mmol/L to mg/dL using the conversion factor 1 mmol/L = 38.67 mg/dL. Thus, cholesterol concentration (mg/dL) was calculated as mmol/L × 38.67.

### 2.6. Circulating Levels of Inflammation

Plasma C-reactive protein (CRP) concentrations were measured using the Rat C-Reactive Protein ELISA Kit (Catalog No. 80670) according to the manufacturer’s instructions. The assay range was 6.25–200 ng/mL. Plasma samples were initially diluted 1:12,000 according to the manufacturer’s protocol. Samples that remained above the upper limit of the assay range were further diluted to 1:24,000 and reanalyzed. The measured concentrations were multiplied by the corresponding dilution factors of 12,000 or 24,000 to determine the CRP concentrations in the original plasma samples. The resulting concentrations were then converted from ng/mL to mg/mL by dividing the values by 1,000,000.

Plasma tumor necrosis factor-alpha (TNF-α) concentrations were quantified using the Invitrogen Rat TNF-α ELISA Kit (Catalog No. KRC3011) according to the manufacturer’s instructions. The kit had an assay range of 11.7–750 pg/mL with an analytical sensitivity of <4 pg/mL. Samples with concentrations exceeding 750 pg/mL were diluted 1:2 with the appropriate assay diluent and reanalyzed. Concentrations obtained from diluted samples were multiplied by the corresponding dilution factor. Values below 11.7 pg/mL were reported as below the assay range.

Plasma interleukin-6 (IL-6) concentrations were measured using the Rat IL-6 Uncoated ELISA Kit (Invitrogen, Thermo Fisher Scientific, Cat. No. 88-50625-88; Lot No. 386398-002) according to the manufacturer’s instructions. A standard curve was generated with concentrations ranging from 2000 to 31.25 pg/mL and a zero standard (0 pg/mL) serving as the blank. The manufacturer’s stated standard curve range was 31–2000 pg/mL. Plasma IL-6 concentrations were determined by interpolation from the standard curve.

### 2.7. Statistical Analysis

Measurements were performed in duplicate, and the average of the duplicate measurements for each treatment group was used for statistical analysis. Results are presented as the mean ± standard error of the mean (SEM). The assumptions of normality and homogeneity of variance were assessed before conducting inferential statistical analyses to determine the appropriate tests for evaluating differences among treatment groups. Normally distributed data that met the assumptions for parametric analysis were analyzed using one-way analysis of variance (ANOVA), followed by Tukey’s post hoc test for multiple pairwise comparisons when a significant group effect was observed. Non-normally distributed data were analyzed using the Kruskal–Wallis test, followed by Dunn’s post hoc test for multiple pairwise comparisons. Weekly body weight trajectories, daily feed intake, daily energy intake, and TNF-α concentrations were analyzed using descriptive statistics only, and no inferential statistical testing was performed for these outcomes. TNF-α was analyzed descriptively as a high proportion of values fell below the lower limit of the assay range (11.7 pg/mL). For feed and energy intake, feed consumption was measured at the cage level; therefore, the cage rather than the individual animal was the experimental unit. Final body weight was analyzed using analysis of covariance (ANCOVA), with treatment group as the fixed factor and baseline body weight as the covariate. Baseline-adjusted final body weights were obtained from the ANCOVA and are presented as estimated marginal means ± standard error (SE). Confidence intervals were calculated at the 95% confidence level. Statistical significance was set at *p* < 0.05.

## 3. Results

### 3.1. Daily Feed Intake

The mean daily feed intake per cage for the control, 4% blueberry powder, and 4% blueberry extract groups was 66.28 ± 03, 62.92 ± 2.6, and 67.52 ± 2.1 g/cage/day, respectively. Similarly, the mean feed intake for the 4% collard greens powder, 4% collard greens extract, and combined 2% blueberry powder and 2% collard greens powder groups was 65.72 ± 3.1, 70.16 ± 2.6, and 64.94 ± 0.5 g/cage/day, respectively. In the semaglutide group, the mean feed intake was 48.30 ± 2.3 g/cage prior to mortality, when animals were housed two per cage. Following the death of five animals during the third week, the three surviving animals were individually housed. Thereafter, the mean feed intake among the surviving animals was 20.4 ± 0.3 g/cage/day, with one rat per cage.

### 3.2. Daily Energy Intake

The mean daily energy intake per cage for the control, 4% blueberry powder, and 4% blueberry extract groups was 265.12 ± 0.15, 239.10 ± 0.8, and 256.58 ± 0.6 kcal/cage/day, respectively. Similarly, the mean energy intake for the 4% collard greens powder, 4% collard greens extract, and combined 2% blueberry powder and 2% collard greens powder groups was 249.74 ± 6.1, 266.61 ± 0.75, and 246.77 ± 0.8 kcal/cage/day, respectively. In the semaglutide group, the mean energy intake was 193.20 ± 0.3 kcal/cage/day prior to mortality. Following the death of five animals during the third week, and subsequent individual housing of the three surviving animals, the mean energy intake was 81.60 ± 0.32 kcal/cage/day, with one rat per cage.

### 3.3. Body Weight

**Figure 1:**
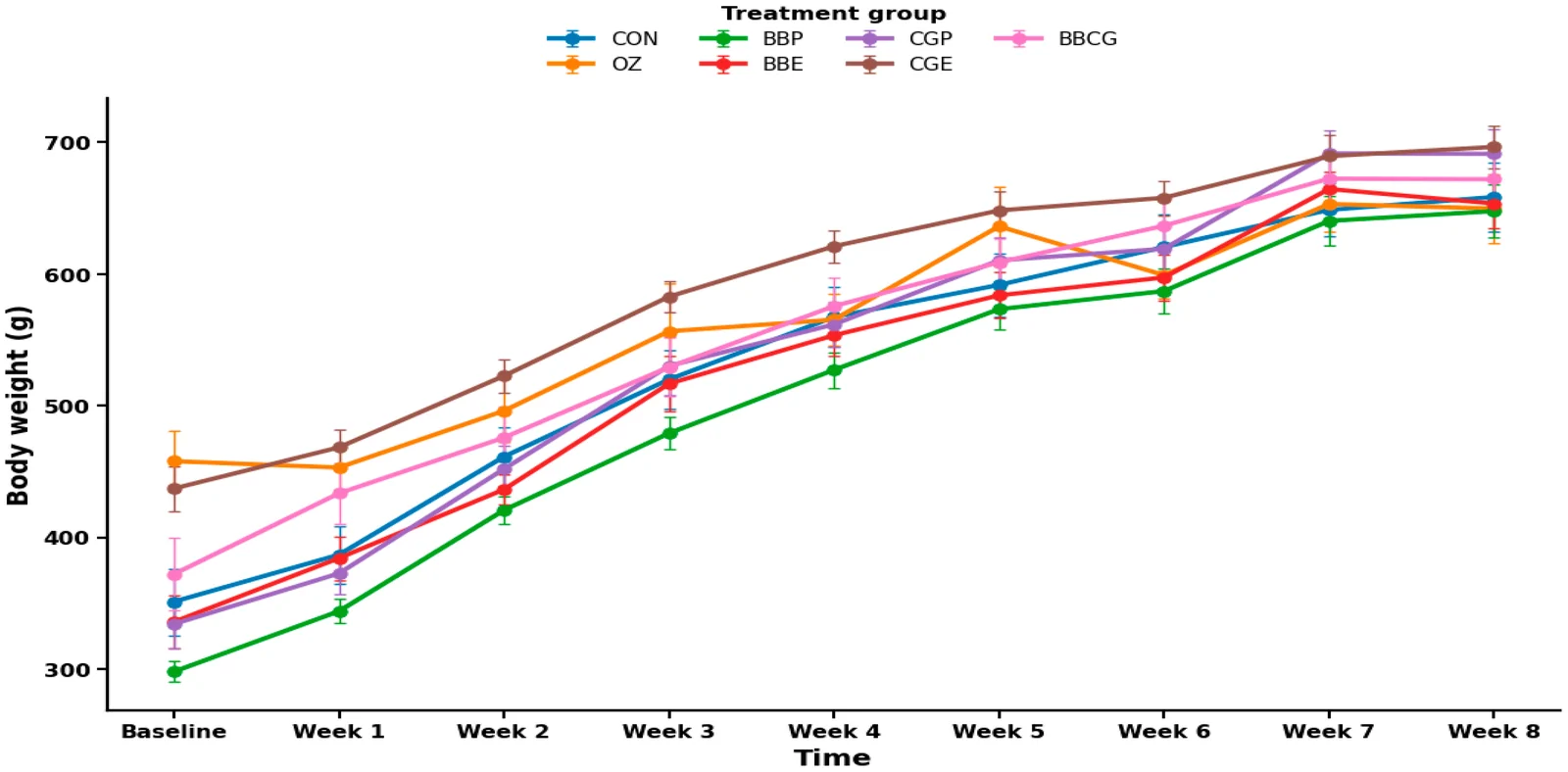
Observed body weight trajectories of Zucker rats following the 8 weeks of dietary intervention. Values are presented as the mean ± SEM (standard error of mean)**;** no inferential statistical comparisons were performed for the longitudinal body weight data. CON, control; OZ, Ozempic (0.25 mg/kg body weight/week); BBP, blueberry powder (4% diet); BBE, blueberry extract (4% diet); CGP, collard greens powder (4% diet); CGE, collard greens extract (4% diet); BBCG, blueberry + collard greens mixture (2% each in diet).

Figure 1 shows the observed body weight trajectories of Zucker obese rats during the 8-week dietary intervention. Mortality in the semaglutide-treated group resulted in a final sample size of *n* = 3 in the positive control group. From the results, body weight increased across all treatment groups.

Table 2 summarizes the average, baseline body weight, final body weight, and baseline-adjusted final body weight. Due to baseline body weight differences among treatment groups, final body weight was analyzed using ANCOVA, with baseline body weight included as the covariate and treatment group as the fixed factor. After adjustment for baseline body weight, no significant treatment effect on final body weight was observed (F(6, 43) = 1.097, *p* = 0.380, partial η^2^ = 0.133).

### 3.4. Plasma Blood Glucose

**Figure 2:**
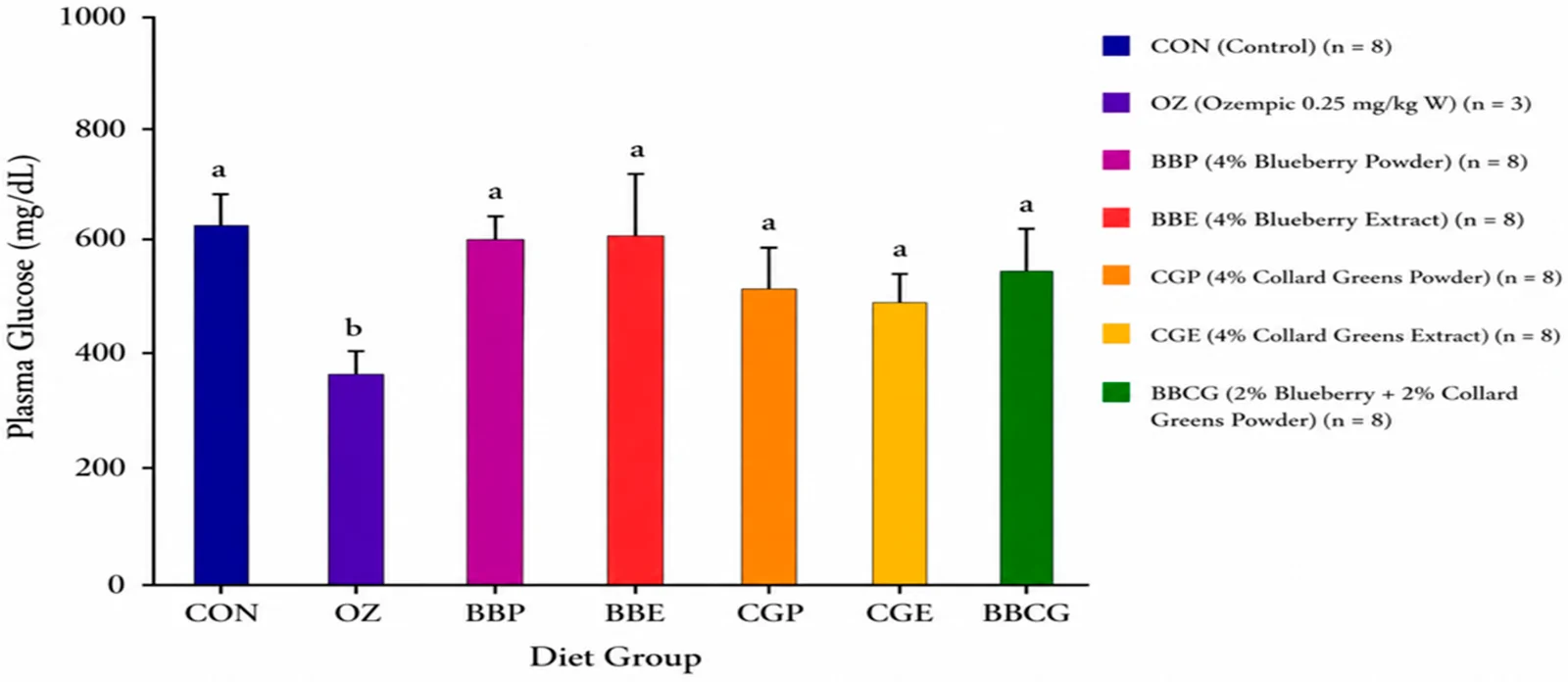
Impact of dietary intervention on plasma glucose concentration. Values are presented as the mean ± SEM (standard error of mean). ^ab^ Different superscript letters within the same row indicate significant differences among groups (*p* < 0.05).

Figure 2 summarizes the effect of dietary intervention, control diet and positive control diet (Ozempic-treated groups) on the plasma glucose concentration of Zucker obese rats. Mortality in the Ozempic-treated group resulted in a final sample size of three rats (*n* = 3) for statistical analysis. All diet-treated groups exhibited lower plasma glucose concentrations than the control group; however, these reductions were not statistically significant (*p* > 0.05). The Ozempic-treated group exhibited significantly lower plasma glucose concentrations than the control and diet-treated groups (*p* < 0.05).

### 3.5. Lipid Profile

Table 3 summarizes the effects of dietary interventions on the lipid profile of Zucker obese rats. Mortality in the Ozempic-treated group resulted in a final sample size of three rats (*n* = 3) for statistical analysis and reduced the statistical power in this group. No significant differences were observed in total cholesterol concentrations among the treatment groups (*p* > 0.05). In contrast, significant differences were observed in HDL-C and LDL-C concentrations (*p* < 0.05). The blueberry and collard greens combination group had the highest HDL-C concentration (89.79 ± 3.61 mg/dL), followed by the 4% collard greens extract (81.44 ± 2.12 mg/dL) and 4% collard greens powder (81.20 ± 1.84 mg/dL) groups. Rats fed the 4% blueberry powder and 4% blueberry extract diets exhibited HDL-C concentrations of 65.98 ± 2.05 mg/dL and 65.07 ± 2.54 mg/dL, respectively. These values were significantly higher than those observed in the control (24.10 ± 1.68 mg/dL) and Ozempic (19.98 ± 0.60 mg/dL) groups (*p* < 0.05).

Rats fed with the combined blueberry and collard greens diet, the 4% collard greens extract diet, and the 4% collard greens powder diet exhibited the lowest plasma LDL cholesterol concentrations, with no significant differences among these groups (*p* > 0.05). The 4% blueberry powder and 4% blueberry extract diets also significantly reduced LDL-C concentrations compared to the control group (*p* < 0.05). The Ozempic-treated (OZ) group did not differ significantly from the control group (*p* > 0.05).

### 3.6. Circulating Levels of Inflammation

Table 4 summarizes the effects of dietary interventions on plasma IL-6 and C-reactive protein concentrations of Zucker obese rats. Mortality in the Ozempic-treated group resulted in a final sample size of three rats (*n* = 3) for statistical analysis and reduced the statistical power in this group.

Significant differences were observed in plasma IL-6 and C-reactive protein (CRP) concentrations among the treatment groups (*p* < 0.05). The Ozempic-treated and 4% collard greens extract groups exhibited the lowest plasma IL-6 concentrations (36.00 ± 0.10 and 34.65 ± 3.90 pg/mL, respectively), which were significantly lower than that of the control group (339.57 ± 185.65 pg/mL *p* < 0.05). The 4% blueberry powder, 4% blueberry extract, 4% collard greens powder, and blueberry and collard greens combination groups also exhibited lower IL-6 concentrations than the control group; however, these differences were not statistically significant (*p* > 0.05). The mean IL-6 concentrations recorded in these groups fell within the manufacturer’s stated standard curve range of 31–2000 pg/mL.

#### TNF-α

TNF-α concentrations were detectable in only two groups. The mean TNF-α concentrations were 373.32 ± 54.47 pg/mL in the control group and 14.61 ± 10.02 pg/mL in the 4% collard greens extract group. TNF-α concentrations in all other treatment groups were below the lower limit of the assay range (11.7 pg/mL).

## 4. Discussion

Body weight was assessed as an indicator of the rats’ response to dietary intervention. From the results, all treatment groups gained weight during the eight-week feeding period (Figure 1). Due to differences in baseline body weight among the treatment groups, final body weight was adjusted for baseline body weight using ANCOVA. After adjustment, no significant differences were observed in final body weight among the treatment groups (*p* = 0.380). Although the Ozempic-treated group had the lowest adjusted final body weight, the difference was not statistically significant. These findings suggest that neither the dietary treatments nor semaglutide significantly affected final body weight after accounting for baseline differences. Rats fed with blueberry powder and collard greens diet continued to gain weight, similar to the control group (Table 2). Although blueberry powder and collard green powder are rich in bioactive compounds such as polyphenols, flavonoids, and antioxidants, these compounds did not produce a reduction in body weight during the study period. It is possible that the amounts of blueberry and collard greens and the short duration of the study (8 weeks) were not sufficient to produce a stronger effect on body weight. This suggests that blueberry’s metabolic effects on weight may be subtler and may require a longer intervention period to influence body weight regulation.

A high mortality rate was observed in the Ozempic-treated group (semaglitude), where five animals died during the third week of the experiment. Although the exact cause of death was not confirmed, reduced fluid intake and dehydration associated with treatment-related gastrointestinal side effects and appetite suppression were considered plausible factors that may have contributed to the observed mortality [36]. The remaining three animals in the group received subcutaneous injections of fluids and survived until the end of the study.

Plasma glucose concentration was used as an indicator of glycemic control across the experimental groups. Results showed that the Ozempic-treated group exhibited significantly lower plasma glucose levels compared to the control group, although it had a lower statistical power. This indicates that Ozempic^®^ was effective in improving glycemic control in the obese Zucker rat model. The observed reduction in plasma glucose in the positive control group is consistent with the known mechanism of action of semaglutide, a GLP-1 receptor agonist. Semaglutide enhances glucose-dependent insulin secretion, suppresses glucagon release, and improves overall glucose homeostasis [37]. These combined effects contribute to improved glycemic regulation independent of weight loss, supporting its established role in the management of metabolic disorders. All dietary interventions reduced plasma glucose concentrations compared to the control group. Reductions in glucose concentration were observed in the collard greens powder, collard greens extract, combined blueberry and collard greens, blueberry, and blueberry extract groups. However, these reductions were not statistically significant. A longer intervention period may be needed to investigate the effects of these dietary interventions on glucose metabolism.

Analysis of plasma lipid profiles indicated that the blueberry and collard greens-based diets increased HDL cholesterol and reduced LDL cholesterol concentrations compared to the control group and those that received Ozempic. Feldman et al. (2021) reported that polyphenols in fruit and vegetables improve dyslipidemia [38]. Polyphenols have also been shown to improve reverse cholesterol transport, thereby increasing circulating HDL while reducing LDL levels [39]. However, because anthocyanin concentrations were not determined in the blueberry-based diet, their potential contribution to the observed lipid changes remains a plausible hypothesis. Similarly, collard greens are known to be rich in bioactive compounds such as glucosinolates and flavonoids [26]. Glucosinolates and their bioactive metabolites have been reported to regulate cholesterol homeostasis by enhancing bile acid synthesis and excretion, reducing intestinal cholesterol absorption, and improving hepatic cholesterol metabolism [40]. These reported mechanisms may provide a plausible explanation for the lipid profile changes observed in the collard greens groups. The extract groups (BBE and CGE) showed no significant differences compared with the corresponding powders (BBP and CGP) in improving HDL and reducing LDL concentrations. The combined blueberry and collard greens diet (BBCG) produced the highest HDL-C concentration and the lowest LDL-C concentration compared to the control group.

Ozempic^®^ (Semaglutide) did not significantly improve HDL and LDL despite its pronounced effects on glycemic control. The lack of statistical significance may have been influenced by the small sample size (*n* = 3) in the group, which reduced the statistical power to detect treatment effects. Additionally, the supportive fluid administration provided to the surviving rats may have influenced the measured lipid concentration. HamaSalih et al. (2024) similarly reported that semaglutide treatment did not significantly increase HDL-C concentrations in Wistar albino rats [41].

The control group exhibited the highest IL-6 concentration, while lower concentrations were observed in the treatment groups. All treatment groups, including Ozempic, showed reduced IL-6 concentrations compared to the control group. However, among the fruit and vegetable-based dietary intervention, only the collard greens extract group showed a statistically significant reduction in IL-6 compared to the control. The reduction in IL-6 observed in the collard greens extract group may be hypothetically explained by the presence of polyphenols and glucosinolate-derived metabolites, which may modulate inflammatory signaling pathways, including NF-κB [42]. Similar findings were reported by Johnson et al. (2019), who showed that diets containing collard greens improved inflammatory status in rats [43]. The authors reported that the antioxidant and anti-inflammatory properties of collard greens reduced oxidative stress and suppressed inflammatory signaling, resulting in lower inflammatory responses [43]. The positive group (Ozempic) also exhibited a significant reduction in IL-6 concentrations compared with the control. This finding may be attributed to the anti-inflammatory effects of semaglutide, a GLP-1 receptor agonist that improves metabolic homeostasis and reduces obesity-associated inflammation [44].

TNF-α concentrations were below the assay’s lower limit of quantification (<11.7 pg/mL) in all groups except the blueberry extract diet and the control diet. The non-detectable TNF-α concentrations may reflect low circulating levels of TNF-α at the time of sample collection. This may be partly due to the transient nature of TNF-α, as its circulating concentration can vary depending on the timing of the inflammatory response and sample collection [45]. TNF-α concentrations were 373.32 ± 54.47 pg/mL and 14.61 ± 10.02 pg/mL in the control and BBE groups, respectively. Given the high proportion of values below the assay’s quantitative range, TNF-α data were analyzed descriptively, and no statistical conclusions regarding treatment effects were drawn. However, a study by Wang et al. (2024) reported significant reductions in inflammatory biomarkers, including TNF-α and IL-6, following blueberry supplementation [46]. The present study observed reductions in IL-6 and CRP in blueberry-treated groups, supporting the anti-inflammatory potential of blueberry.

All treatment groups, including Ozempic, had lower CRP levels compared to the control group. Within the blueberry treatments, both the powder and extract groups showed reductions in CRP, with no statistically significant difference between them. Similarly, no significant differences were observed between collard greens powder and collard greens extracts. The combined blueberry and collard greens group recorded the lowest CRP level. This observation suggests that the combined dietary formulation warrants further investigation for its potential role in modulating inflammatory responses. The reduction in inflammatory biomarkers observed in the Ozempic group is consistent with the known anti-inflammatory effects of semaglutide [44]. As a glucagon-like peptide-1 receptor agonist (GLP-1RA), semaglutide reduces systemic inflammation through both direct and indirect mechanisms suppressing inflammatory signaling pathways, including nuclear factor-kappa B (NF-κB), resulting in decreased production of pro-inflammatory mediators such as IL-6 and C-reactive protein (CRP) [44]. However, the small sample size (*n* = 3) and supportive fluid administration to the surviving rats may have influenced these findings, limiting their interpretation.

## 5. Conclusions

Obesity is a major public health concern associated with metabolic dysregulation and chronic inflammation. Therefore, identifying sustainable and cost-effective dietary strategies to improve obesity-associated metabolic disturbances remains a public health priority. The present study evaluated the effects of blueberry and collard greens-based dietary interventions on selected biomarkers of obesity in obese Zucker rats. The findings revealed that phytochemical-rich diets improved lipid profile and inflammatory status, as evidenced by increased HDL-C, reduced LDL-C, and reductions in inflammatory biomarkers. These preliminary findings warrant further investigation into the potential use of blueberry and collard greens-based dietary interventions for managing obesity-associated metabolic and inflammatory disturbances. The use of only male Zucker obese rats limits the generalizability of the findings, as females may exhibit different metabolic and inflammatory responses. Furthermore, the eight-week intervention period limits conclusions regarding the long-term effects of the dietary treatments. Future studies should include both sexes, longer intervention periods, and human clinical trials to determine the translational relevance of these findings.

**Limitation:** The high mortality observed in the Ozempic-treated group, in which five of the eight rats died, limited the interpretation of findings and comparisons involving the diet and Ozempic-treated group.

## Figures and Tables

**Table 1:** Nutritional composition of experimental diets.

Ingredient	AIN-93	4% BBP	Experimental Group 4% BBE	4%CGP	4%CGE	2%BBP + 2%CGP
Protein (gm%)	20	19	19	19	19	19
Carbohydrates (gm%)	64	61	61	61	61	61
Fat (gm%)	7	7	7	7	7	7
Kcal/gm	4.0	3.8	3.8	3.8	3.8	3.8
Casein (gm)	200	200	200	200	200	200
L-Cysteine (gm)	3	3	3	3	3	3
Corn starch (gm)	397.486	397.486	397.486	397.486	397.486	397.486
Maltodextrin (gm)	132	132	132	132	132	132
Sucrose (gm)	100	100	100	100	100	100
Cellulose (gm)	50	50	50	50	50	50
Soyabean oil (gm)	70	70	70	70	70	70
tBHQ (gm)	0.014	0.014	0.014	0.014	0.014	0.014
Mineral Mix (gm)	35	35	35	35	35	35
Vitamin Mix (gm)	10	10	10	10	10	10
Choline Bitrate (gm)	2.5	2.5	2.5	2.5	2.5	2.5
Blueberry powder (gm)	0	41.7	0	0	0	20.85
Collard greens powder (gm)	0	0	0	41.7	0	20.85
Blueberry extract (gm)	0	0	41.7	0	0	0
Collard greens extract (gm)	0	0	0	0	41.75	0
FD&C Blue Dye 1 (gm)	0	0.05	0	0	0.025	0.0
FD&C Yellow Dye 5 (gm)	0	0	0.025	0.05	0.025	0.0
FD&C Dye 40	0	0	0.025	0	0	0.05
Total	1041.75	1041.75	1041.75	1041.75	1041.5	1041.5

4% BBP, modified AIN-93 with 4% blueberry powder; 4% BBE, modified AIN-93 with 4% blueberry extract; 4% CP, modified AIN-93 with 4% collard greens powder; 4% CGE, modified AIN-93 with 4% collard greens extract; 2%BBP + 2%CGP, modified AIN-93 with 2% blueberry powder and 2% collard greens powder.

**Table 2:** Impact of dietary interventions on body weight outcomes.

Diet Group (*n*)	Baseline Weight (g)	Final Weight (g)	Baseline-Adjusted Final Body Weight (g)
CON (*n* = 8)	351.05 ± 25.37	658.43 ± 26.39	662.65 ± 18.63 ^a^
OZ (*n* = 3)	457.79 ± 23.1	649.63 ± 25.88	607.06 ± 33.20 ^a^
BBP (*n* = 8)	297.90 ± 7.98	647.74 ± 20.39	675.26 ± 20.53 ^a^
BBE (*n* = 8)	335.72 ± 20.40	653.46 ± 18.51	664.41 ± 23.24 ^a^
CGP (*n* = 8)	334.08 ± 18.22	691.21 ± 19.06	702.88 ± 18.95 ^a^
CGE (*n* = 8)	437.00 ± 17.02	696.47 ± 21.48	663.02 ± 21.39 ^a^
BBCG (*n* = 8)	371.97 ± 27.61	671.90 ± 21.48	666.95 ± 18.65 ^a^

Values for baseline body weight, and final body weight for each treatment group are presented as the mean ± SEM (standard error of the mean). Baseline-adjusted final body weight is presented as the estimated marginal mean (EMM) ± SE and was determined using ANCOVA, with baseline body weight as the covariate and treatment group as the fixed factor. The ANCOVA showed no significant treatment effect on baseline-adjusted final body weight (*p* = 0.380). ^a^ Superscript letters within the same row indicate significant differences among groups.(*p* < 0.05) CON, control; OZ, Ozempic (0.25 mg/kg body weight/week); BBP, blueberry powder (4% diet); BBE, blueberry extract (4% diet); CGP, collard greens powder (4% diet); CGE, collard greens extract (4% diet); BBCG, blueberry + collard greens mixture (2% each in diet).

**Table 3:** Impact of dietary interventions on lipid profile.

Diet Group (*n*)	Total Cholesterol (mg/dL)	HDL (mg/dL)	LDL (mg/dL)
CON *(n* = 8)	389.10 ± 29.82 ^a^	24.10 ± 1.68 ^d^	332.48 ± 55.61 ^a^
OZ (*n* = 3)	409.64 ± 1.36 ^a^	19.98 ± 0.6 ^d^	386.88 ± 98.99 ^a^
BBP (*n* = 8)	371.16 ± 28.70 ^a^	65.98 ± 2.05 ^c^	170.79 ± 54.15 ^b^
BBE (*n* = 8)	376.38 ± 26.10 ^a^	65.07 ± 2.54 ^c^	269.93 ± 73.68 ^b^
CGP (*n* = 8)	336.32 ± 39.89 ^a^	81.20 ± 1.84 ^b^	111.46 ± 19.43 ^c^
CGE (*n* = 8)	341.73 ± 23.81 ^a^	81.44 ± 2.12 ^b^	102.34 ± 18.44 ^c^
BBCG (*n* = 8)	356.61 ± 12.76 ^a^	89.79 ± 3.61 ^a^	98.82 ± 28.21 ^c^

Values are presented as the mean ± SEM (standard error of mean). ^abcd^ Different superscript letters within the same row indicate significant differences among groups (*p* < 0.05). CON, control; OZ, Ozempic (0.25 mg/kg body weight/week); BBP, blueberry powder (4% diet); BBE, blueberry extract (4% diet); CGP, collard greens powder (4% diet); CGE, collard greens extract (4% diet); BBCG, blueberry + collard greens mixture (2% each in diet).

**Table 4:** Effect of dietary intervention on inflammatory biomarkers.

Diet Group (*n*)	IL-6 (pg/mL)	CRP (mg/mL)
CON (*n* = 8)	339.57 ± 185.65 ^a^	4.03 ± 0.13 ^a^
OZ (*n* = 3)	36.00 ± 0.1 ^b^	2.67 ± 0.09 ^b^
BBP (*n* = 8)	128.19 ± 51.3 ^a^	2.72 ± 0.07 ^b^
BBE (*n* = 8)	77.4 ± 14.33 ^a^	2.81 ± 0.01 ^b^
CGP (*n* = 8)	153.75 ± 81.55 ^a^	2.77 ± 0.01 ^b^
CGE (*n* = 8)	34.65 ± 3.90 ^b^	2.78 ± 0.03 ^b^
BBCG (*n* = 8)	105.31 ± 63.76 ^a^	0.88 ± 0.13 ^c^

Values are presented as the mean ± SEM (standard error of mean). Different superscript letters within the same row indicate significant differences among groups (*p* < 0.05). CON = control group; OZ = Ozempic (0.25 mg/kg body weight/week); BBP = blueberry group; powder (4% diet); BBE = blueberry extract (4% diet); CGP = collard greens powder (4% diet); CGE = collard greens extract (4% diet); BBCG = blueberry + collard greens mixture (2% each in diet).

## Data Availability

Data are available upon request from the corresponding author.
